# Ecological Aspects of Phlebotomine Sandflies (Diptera: Psychodidae) from a Cave of the Speleological Province of Bambuí, Brazil

**DOI:** 10.1371/journal.pone.0077158

**Published:** 2013-10-09

**Authors:** Gustavo Mayr de Lima Carvalho, Reginaldo Peçanha Brazil, Mariana Campos das Neves Farah Ramos, Paula Cavalcante Lamy Serra e Meira, Ana Paula Lusardo de Almeida Zenóbio, Helbert Antônio Botelho, Cristiani de Castilho Sanguinette, Lara Saraiva, José Dilermando Andrade Filho

**Affiliations:** 1 Grupo de Estudos em Leishmanioses, Centro de Pesquisas René Rachou, Fundação Oswaldo Cruz, Belo Horizonte, Minas Gerais, Brazil; 2 Departamento de Fisiologia e Bioquímica de Insetos, Instituto Oswaldo Cruz, Fundação Oswaldo Cruz, Rio de Janeiro, Rio de Janeiro, Brazil; Centro de Pesquisa Rene Rachou/Fundação Oswaldo Cruz (Fiocruz-Minas), Brazil

## Abstract

Phlebotomines are invertebrate hosts of *Leishmania* genus species which are etiological agents of leishmaniases in humans and other mammals. Sandflies are often collected in entomological studies of caves both in the inner area and the adjacent environments. Caves are ecotypes clearly different from the external environment. Several caves have been opened to public visitation before any studies were performed and the places do not have scientific monitoring of the fauna, flora, geological and geographical characteristics. These events can lead to the loss of geological and biological information. Considering these aspects, this study aimed to describe the sand fly fauna, including the ecological features, in a limestone cave at the Speleological Province of Bambuí (Minas Gerais State, Brazil). A total of 8,354 specimens of sandflies belonging to 29 species were analyzed: *Lutzomyia cavernicola* (20%), *Nyssomyia intermedia* (15%), *Martinsmyia oliveirai* (13%), *Evandromyia spelunca* (12%), *Evandromyia sallesi* (11%), *Migonemyia migonei* (9%), *Nyssomyia whitmani* (9%), *Sciopemyia sordellii* (4%) and *Lutzomyia longipalpis* (2%). The others species represent 5% of the total. This manuscript presents data found on richness, diversity, evenness and seasonality, comparing the sand fly fauna trapped in the cave and its surroundings.

## Introduction

The phlebotomine sandflies (Diptera, Psychodidae) are considered the only natural vectors of *Leishmania* (Kinetoplastida, Trypanosomatidae) genus. These protozoans are the etiological agents of the leishmaniasis, a neglected tropical disease [1,2]. The transmission of the *Leishmania* parasite species to the vertebrate host occurs during the bite of infected female sandflies [3]. The geographical distribution of leishmaniasis has undoubtedly expanded and is now being reported in areas that were previously non-endemic [4].

The resting places of the adult sandflies vary according to the species and to the environmental conditions, such as forest floor, small shrubs and plants, mammal nests and burrows, and rock crevices and caves [5]. In general, the sandflies activity period ranges from dusk to dawn and these insects remain most of the day at rest in natural shelters.

Caves are considered stable environments in comparison with epigean habitats and are also characterized by a permanent lack of light far from entrances [6,7]. Fauna adapted to these conditions can be classified according to their level of adaptation: troglobites (animals that present unique modifications to cave environments), troglophiles (adapted animals, but devoid of modifications that can also develop in the external environment) and trogloxenes (animals that use caves for shelter or refuge) [8].

The caves, probably, can be considered as important geographical barriers, becoming evocative ecotypes for finding new genus and species of sandflies [9-15] and also seems to favor the occurrence of morphological anomalies in these insects [16], becoming important to study the sand fly fauna in this ecotype.

Caves are important for the ecosystem balance in their areas of occurrence. The interferences in the physical environment caused by human actions or natural phenomena are directly reflected in caves located in areas in which these impacts can occur. The alteration of the original structure of a cave system caused by different impacts can disturb the external system, stressing the state of unbalance of a given ecosystem [17-19].

Thus, considering that the sandflies are found in the cave environment and that this environment can sometimes offer attractive tourist and economic for the man, the chances of transmission of leishmaniasis may increase, considering not only possible changes, but the mere chance of contact of these insects with the people that visit this environment, which is still little studied. Recently was demonstrated that the environmental changes can cause the expansion of leishmaniases through closer contact between man and the vector of this disease [20].

There are few studies on the phlebotomine fauna in caves in Brazil. The recent expansion of ecotourism and the lack of management programs to access these environments may lead to the loss of valuable geological, paleontological or biological information due to predatory human exploitation of caves.

Some studies pointed out that insect diversity and density of sandflies in caves can be equal to or greater than those found in the forest [21,22]. Based on this context, and on the medical importance of phlebotomines sandflies, this work aimed to study the sandflies fauna in a cave and its surroundings area, considering the ecological aspects of these insects and comparing the fauna of both ecotypes. The cave belongs to the Speleological Province of Bambuí and is situated in the municipality of Lassance (Minas Gerais State, Brazil).

## Material and Methods

### Ethics Statement

Collections were made in a cave situated on a private farm. A term of consent was established to run the captures in this cave.

### Study area

This study was conducted in the municipality of Lassance, northern of Minas Gerais State, in the microregion of Pirapora. The municipality covers an area of approximated 3,200 km^2^ and has an estimated population of 6,484 inhabitants [23]. The region has 10 caves registered in the National Register of Caves of Brazil (CNC) [24]. The Speleological Province of Bambuí is partly situated in the southeastern region of Tocantins, central east and southeast of Goiás, central west and north west of Minas Gerais and west of Bahia States. This speleological province is considered the largest in the country, with an extension of 105,200 km^2^ [25]. The cave chosen for this study (coordinates 17°59'40.01"S, -44°39'3.23"W) has not been registered and it is situated in a private farm about 20 km from the center of Lassance municipality. It is a limestone cave with a horizontal extension of approximately 100 meters; the altitude is approximately 700 meters above sea level.

### Sampling of sandflies

Sampling was performed monthly from June 2008 to May 2010, using automatic light traps, model HP [26]. The collection points were patterned with five traps being used inside the cave and five traps in the area around, as it is described below.

The collection points inside the cave were situated within 10 meters of each other, with the first point located 10 meters from the cave entrance (Table 1). The collection points in the cave vicinity were arranged sparsely without a fixed distance among them. This distribution was done considering the environmental variation present in the surrounding area (Table 1). The traps were exposed during two consecutive days, continuously, with a total sampling effort of approximately 40 hours/trap per collection, for instance, a sampling effort of 200 hours inside and outside the cave per month. The temperature and relative humidity were measured using a digital thermo-hygrometer, inside and outside the cave. The averages of the climatic variables were used to correlate with the monthly densities of sandflies.

**Table 1:** Characterization of exposure points of light traps, in catches carried out monthly, between June 2008 and May 2010, Lassance municipality, Minas Gerais State, Brazil.

	**Cave Area**	
**Locations/Traps**		**Characterization of points**
Cave 01		Trap located 10 meters from the cave entrance. Presence of light during the day period.
Cave 02		Trap located 20 meters from the cave entrance. Low penetration of light during the day period.
Cave 03		Trap located 10 meters from the cave 02 point. Total absence of light.
Cave 04		Trap located 10 meters from the cave 03 point. Total absence of light.
Cave 05		Trap located 10 meters from the cave 04 point. Total absence of light.
	**Surrounding Area**	
**Locations/Traps**		**Characterization of points**
Peridomicile		Hen house of a peridomicile. House 400 meters from the cave.
Pasture		Pastureland of cattle, underbrush area. Trap 200 meters from the cave.
Wooded area		Area of sparse vegetation, remnant of deforested area. Trap 100 meters of the cave.
10 meters out of entrance		Area near to the rock formation. Trap 10 meters from the cave entrance.
Cave entrance		Trap located at the entrance of the cave, but outside of it.

Sandflies male were prepared and mounted on slides for identification. The females were dissected and identified based on the spermathecae characteristics. The specimens were identified according to the classification proposed by Galati [27] and the abbreviation of generic names was performed according to Marcondes [28]. The voucher specimens were deposited in the phlebotomine sandfly collection of the Centro de Pesquisas René Rachou/Fiocruz.

### Statistical analyses

Data were organized through Excell 97/2003, which was also used in the descriptive statistics. Graph Pad Instat software was used for statistical analyses. Chi-square test was used to compare the species distribution in the environments (surrounding area and cave) in the overall analysis. The calculations of Shannon and Simpson Diversity indexes, Richness and Equitability J were performed using the program DIVES v 2.0 [29].

## Results

A total of 8,354 specimens were collected, belonging to 10 genera and 29 species. Taking into account the environments separately, 5,255 (63%) sandflies were collected inside the cave and 3,099 (37%) in the adjacencies of the cave (Table 2). Considering the sex ratio, 4,564 (55%) of the specimens were females and 3,790 (45%) males.

**Table 2:** Total of Phlebotomine sandflies species collected between June 2008 and May 2010, with light trap (model HP), according to collection site (Cave or Surrounding), in the municipality of Lassance, Minas Gerais State, Brazil.

**Species**	**Locations of Cave Area**						**Locations of Surrounding Area**					
	Cave 01	Cave 02	Cave 03	Cave 04	Cave 05	**Total (%)**	Hen House	Pasture	Wooded area	10 meters out	Cave entrance	**Total**
*Br. avellari*	3	3	-	1	3	10 (0.19)	2	-	-	1	-	3 (0.10)
*Br. pintoi*	-	-	-	-	-	-	14	-	2	1	-	17 (0.55)
*Ev. bacula*	-	-	-	-	-	-	-	1	-	-	-	1 (0.03)
*Ev. bourrouli*	-	-	-	-	-	-	1	-	-	-	-	1 (0.03)
*Ev. evandroi*	-	-	-	-	1	1 (0.02)	19	11	6	11	1	48 (1.55)
*Ev. lenti*	-	-	-	-	-	-	-	-	1	1	-	2 (0.06)
*Ev. sallesi*	287	161	98	54	115	715 (13.61)	17	7	59	18	66	167 (5.39)
*Ev. spelunca*	416	242	153	78	73	962 (18.31)	2	-	38	2	22	64 (2.07)
*Ev. teratodes*	-	1	-	-	-	1 (0.02)	-	-	-	-	-	-
*Ev. termitophila*	10	-	5	1	1	17 (0.32)	11	9	6	5	6	37 (1.19)
*Lu. cavernicola*	672	370	264	120	125	1551 (29.51)	-	1	31	9	57	98 (3.16)
*Lu. longipalpis*	28	-	1	-	-	29 (0.55)	16	63	9	6	10	104 (3.36)
*Lu. renei*	2	4	1	1	-	8 (0.15)	-	-	-	-	-	-
*Mt. oliveirai*	701	141	37	23	11	913 (17.37)	27	13	53	6	92	191 (6.16)
*Mg. migonei*	390	59	35	20	18	522 (9.93)	28	19	37	20	140	244 (7.87)
*Mi. echinatopharynx*	2	-	1	-	-	3 (0.06)	6	1	-	1	1	9 (0.29)
*Mi. quinquefer*	12	-	-	-	-	12 (0.23)	3	-	23	2	29	57 (1.84)
*Mi. rorotaensis*	-	-	-	-	-	-	-	1	-	-	-	1 (0.03)
*Ny. intermedia*	25	5	4	25	2	61 (1.16)	244	84	351	208	288	1175 (37.92)
*Ny. neivai*	1	-	-	-	-	1 (0.02)	2	-	-	9	5	16 (0.52)
*Ny. whitmani*	51	3	15	16	-	85 (1.62)	145	105	196	93	136	675 (21.78)
*Pa. aragaoi*	-	-	-	-	-	-	-	-	1	-	-	1 (0.03)
*Pa. lutziana*	2	1	-	1	3	7 (0.13)	3	1	1	3	-	8 (0.26)
*Pi. christenseni*	1	-	-	-	-	1 (0.02)	3	3	4	2	1	13 (0.42)
*Pi. misionensis*	-	-	-	-	-	0	-	1	2	-	-	3 (0.10)
*Pi. Monticola*	1	-	1	-	-	2 (0.04)	-	-	11	1	6	18 (0.58)
*Pi. pessoai*	-	-	-	-	-	-	36	12	32	20	24	124 (4.00)
*Sc. microps*	1	-	-	1	2	4 (0.08)	-	-	-	-	-	-
*Sc. sordellii*	197	49	21	44	39	350 (6.66)	2	3	9	2	6	22 (0.71)
**Total**	2802 (53.32)	1039 (19.77)	636 (12.10)	385 (7.33)	393 (7.48)	**5255 (100.00)**	581 (18.75)	335 (10.81)	872 (28.14)	421 (13.59)	890 (28.72)	**3099 (100.00)**

Considering both environments, *Evandromyia* Mangabeira, 1941 was the genus with the highest number of species captured, with eight species. However, the species with the highest numbers of specimens captured were respectively: *Lutzomyia cavernicola* (Costa Lima, 1932) (20%), *Nyssomyia intermedia* (Lutz & Neiva, 1912) (15%), *Martinsmyia oliveirai* (Martins, Silva & Falcão, 1970) (13%), *Evandromyia spelunca* Carvalho, Sanguinette, Brazil & Andrade Filho, 2011 (12%), *Evandromyia sallesi* (Galvão & Coutinho, 1939) (11%). The other species amounted to less than 10% of the total. The analyses of abundance considering each environment separately showed different results. The surrounding area presented two abundant species: *Ny. intermedia* (38%) and *Nyssomyia whitmani* (Antunes & Coutinho, 1939) (22%). The cave presented five species which were abundant: *Lu. cavernicola* (29%), *Ev. spelunca* (18%), *Ma. oliveirai* (17%), *Ev. sallesi* (14%), *Migonemyia migonei* (França, 1920) (10%) (Table 2).

**Figure 1:**
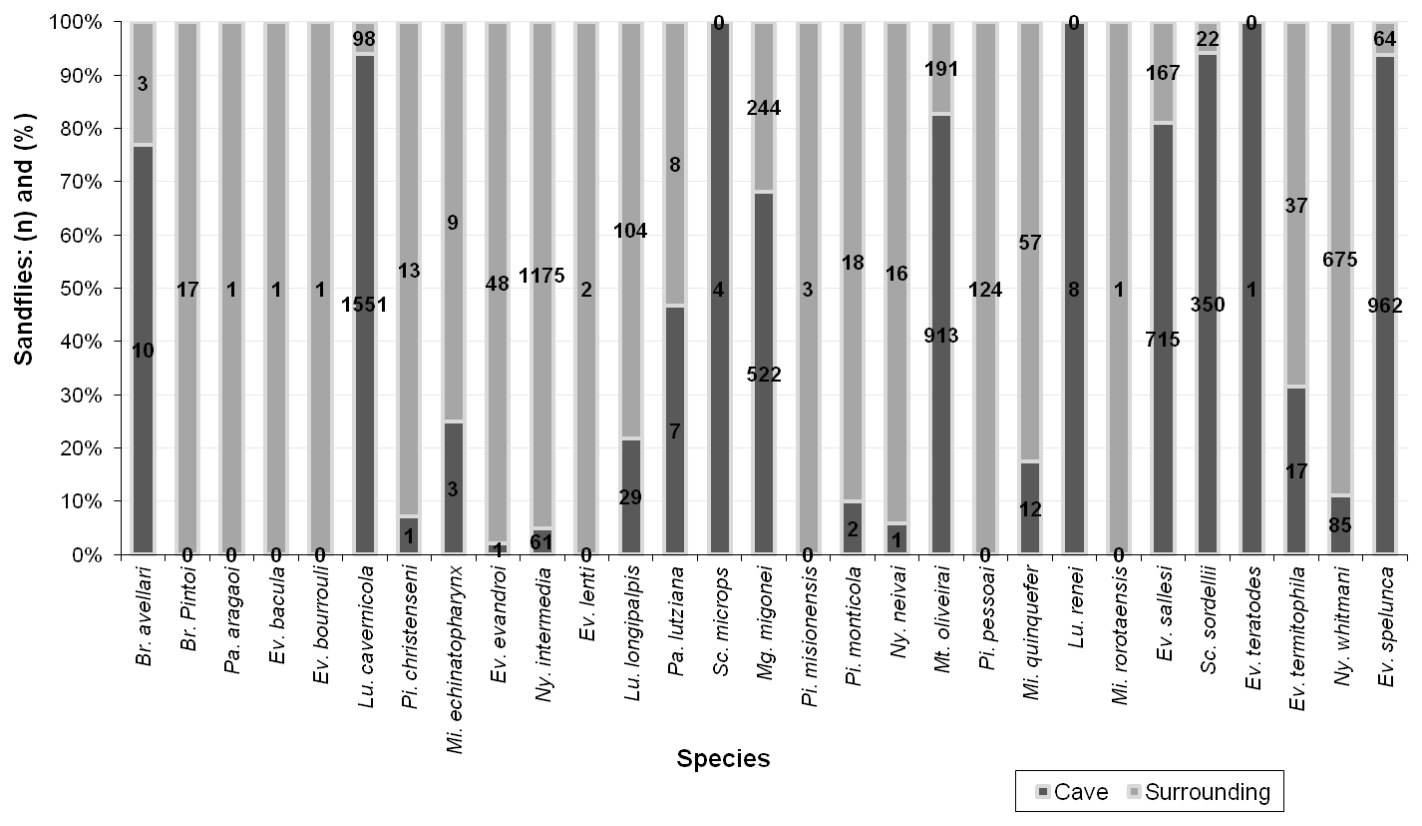
Distribution of the species trapped monthly, with light traps, between June 2008 and May 2010, according to the location: inside the cave and surrounding area – Lassance municipality, Minas Gerais State, Brazil.

The comparison of the number of species collected in each environment revealed that from the total of 29 species, 18 were common to both environments, 26 were recorded in the surrounding area and 21 inside the cave. Only three species were found exclusively in the cave, whereas eight species were collected only in the surrounding area (Figure 1).

A significant number of sandflies were captured around the cave entrance, compared with the other points of collect. The trap “Cave 01” captured 53% of the total of sandflies collected inside the cave and the “cave entrance” trap, 29% of the total sandflies collected outside the cave (Table 2). The sum of the totals collected by these two traps represents 44% of total sandflies captured in both environments.

The analyses of the richness index, Simpson’s diversity, Shannon’s diversity and Jaccard’s evenness are summarized in Table 3. The comparison of the indexes demonstrates that the cave and the surrounding area showed a very similar profile. Considering the Simpson’s index, the cave area (0.9869) revealed greater diversity than the surrounding area (0.9676), whereas the Shannon’s index demonstrated that the surrounding area showed greater diversity (0.893) than the cave area (0.8190). Both areas had similar richness and evenness index (Table 3).

**Table 3:** Indexes of diversity, evenness and richness to the cave area and the surrounding area of the cave in the region of Lassance Municipality from June 2008 to May 2010.

**Ecological index**				
**Cave area**				
**Locations**	**Simpson’s Diversity**	**Shannon’s Diversity**	**J Evenness**	**Richness**
Cave 1	0.9515	0.8275	0.6471	Smax: 37.2 - S2: 1.9
Cave 2	0.9820	0.7245	0.6713	Smax: 9.8 - S2: 1.9
Cave 3	0.9809	0.7090	0.6365	Smax: 7.8 - S2: 1.9
Cave 4	0.9763	0.8335	0.7483	Smax: 5.9 - S2: 1.9
Cave 5	0.9714	0.7234	0.6704	Smax: 9.8 - S2: 1.9
Cave area	0.9869	0.8190	0.6194	Smax: 15.7 - S2: 1.9
**Surrounding area**				
**Locations**	**Simpson’s Diversity**	**Shannon’s Diversity**	**J Evenness**	**Richnees**
Chicken’s house	0.9153	0.8116	0.6347	Smax: 7.8 - S2: 1.9
Wooded area	0.9441	0.8489	0.6525	Smax: 11.8 - S2: 1.9
Cave entrance	0.9707	0.8906	0.7238	Smax: 7.8 - S2: 1.9
10 meters out of entrance	0.9678	0.7593	0.5742	Smax: 5.9 - S2: 1.9
Pasture area	0.9469	0.8302	0.6747	Smax: 2.0 - S2: 1.9
Surrouding area	0.9676	0.8931	0.6312	Smax: 15.7 - S2: 1.9

**Figure 2:**
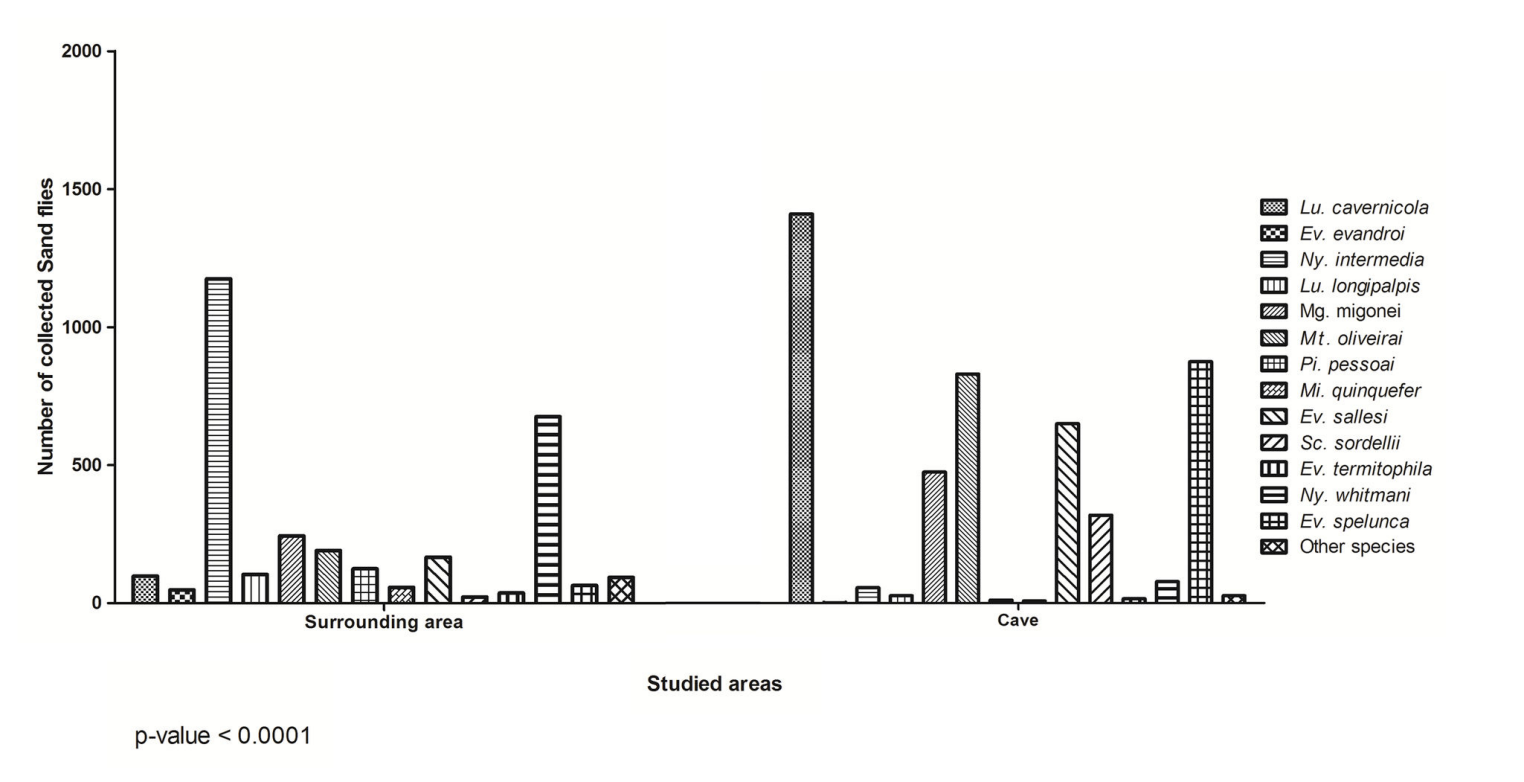
Species or group of species captured monthly between June 2008 and May 2010 in both environments (inside the cave and outside of the cave) with significant differences in the numbers of the specimens. Lassance municipality, Minas Gerais State, Brazil.

Despite the similarities of the cave and surrounding areas, considering the diversity, evenness and richness indexes, the analysis of the faunistic composition considering the abundance of specimens per species demonstrated a statistically significant difference between these areas (Chi-square test, p-value < 0.0001) (Figure 2). It is noteworthy that this comparison also favors the common and abundant species.

The species collected in low numbers, less than or equal to 0.02% of the total collected (*Brumptomyia avellari* (Costa Lima, 1932), *Brumptomyia pintoi* (Costa Lima, 1932), *Evandromyia bacula* (Martins, Falcão & Silva, 1965), *Evandromyia bourrouli* (Barreto & Coutinho, 1941), *Evandromyia lenti* (Mangabeira, 1938), *Evandromyia teratodes* (Martins, Falcão & Silva, 1964), *Lutzomyia renei* (Martins, Falcão & Silva, 1957), *Micropygomyia echinatopharynx* Andrade Filho, Galati, Andrade & Falcão, 2004, *Micropygomyia rorotaensis* (Floch & Abonnenc, 1944), *Nyssomyia neivai* (Pinto 1926), *Pintomyia christenseni* (Young & Duncan, 1994), *Pintomyia misionensis* (Castro, 1959), *Pintomyia monticola* (Costa Lima, 1932), *Psathyromyia aragaoi* (Costa Lima, 1932), *Psathyromyia lutziana* (Costa Lima, 1932), *Siopemyia microps* (Mangabeira, 1942)] were considered together during the analysis.

**Figure 3:**
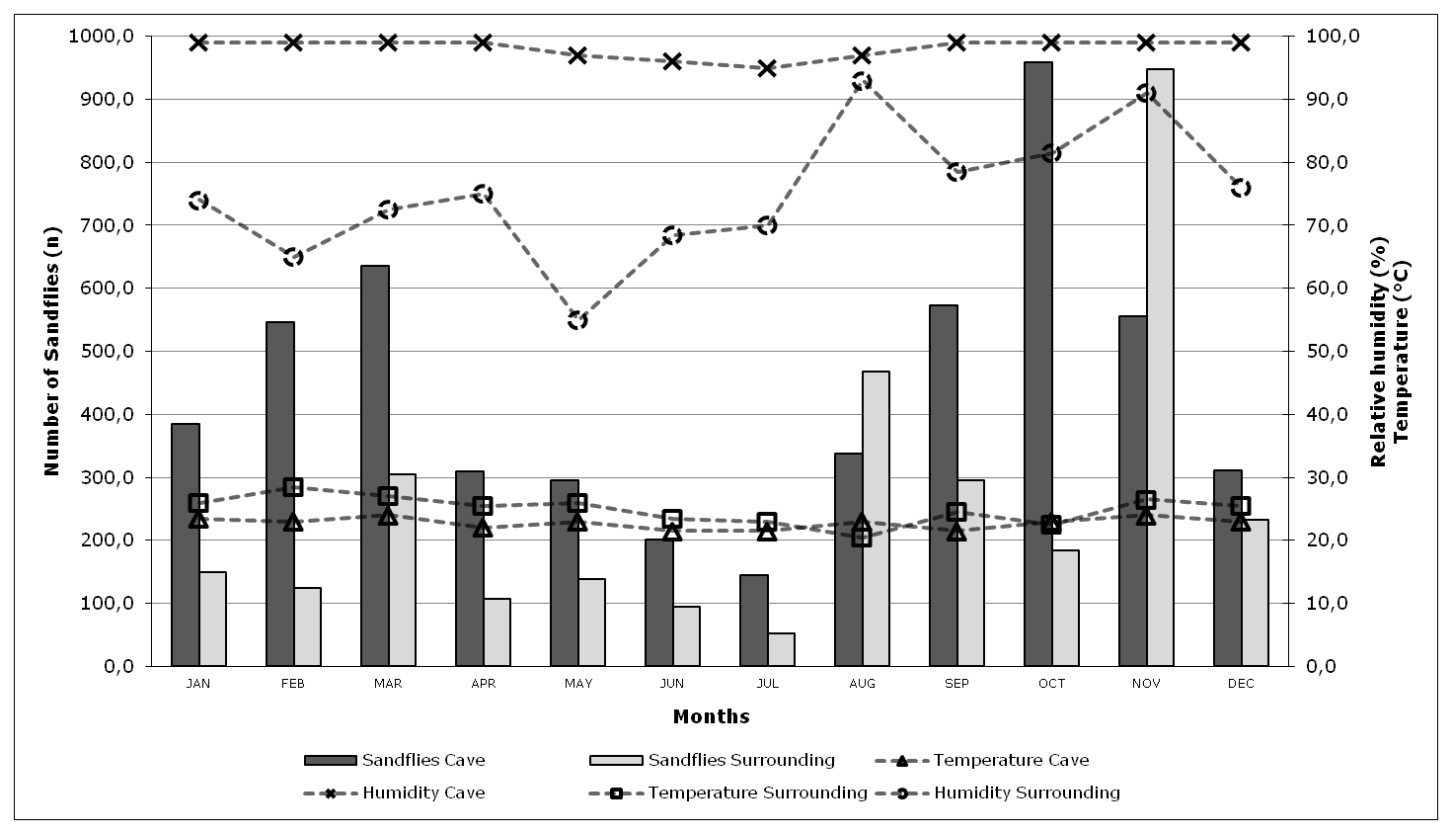
Monthly average of the total of specimens captured between June 2008 and May 2010, the relative humidity and the temperature in the cave and surroundings. Lassance municipality, Minas Gerais State, Brazil.

The points representing the seasonal variation of sandflies collected in both studied areas, and the climatic variables were represented in Figure 3. This analysis concerns the monthly averages of the climate variables recorded, and the monthly total of sandflies collected.

Regarding the climatic variables registered inside the cave and in the surrounding area, it can be noticed clearly that there were virtually no variation within the cave. These data indicate that the environment inside the cave tends to be stable during all seasons, with a maximum variation of 2°C in temperature along the year and the relative humidity around 99%. The temperature registered in the surrounding area varied up to 8°C throughout the year. The relative humidity varied from 55% in drier periods to 93%, in rainy periods. Even though the temperature and relative humidity presented large daily amplitudes in the surrounding environment, it was not observed in the cave environment data.

Comparing both environments, a higher density of sandflies was observed in the cave than in the surrounding area, all the months of the study, except August and November (Figure 3).

## Discussion

Chagas Disease was discovered in the municipality of Lassance [30]. Due to this fact, several collections of insects were done at this location from 1920 to 1935. Some species of sandflies from this region were described, including *Psathyromyia aragaoi* (Costa Lima 1932) and *Brumptomyia pintoi* (Costa Lima 1932), which are present in our samples.

The present work recorded 29 species belonging to 10 genera. Comparing the species captured in previous studies, four species registered by Saraiva et al. [31] were not registered in our study. Until now, 35 species of sandflies were registered in the municipality of Lassance. This number is fairly representative, regarding the fauna of Minas Gerais state, which comprises 93 species, belonging to 16 genera [32].

Lutzomyiina was the predominant subtribe, and the genus that had the largest number of collected specimens was *Evandromyia*. This fact was also recorded in the States of Tocantins and Mato Grosso do Sul, Brazil [33,34]. This group is quite common in the Brazilian savannah (*cerrado*), including the Lassance region [31]. Andrade & Dantas-Torres [32] pointed this genus as the most representative of the Minas Gerais state, accounting 16 species. In our study, this genus was represented by eight species.

Some sandflies are often associated with forest environments. They are also found in areas without continuous vegetation cover, including urban areas and caves [35]. Approximately 260 sand fly species are recorded in Brazil, and 84 of these [36] have already been registered from caves [10,11,13,21,22,37-42]. It is important to report that at least 15 species has its type locality from caves, including species classified as troglobites [10], with adaptations that allow them to live exclusively in this ecotype.

Considering the percentage and the number of species collected in the surrounding area and in the cave, 63% of sandflies were trapped in the cave, and 37% were captured in the surrounding. A total of 21 species have been reported inside the cave, and 26 species have been found in the surrounding area. These data reveal that the cave environment may prove as an important refuge for sandflies. The majority of the species collected are classified as troglophiles. However, the presence of trogloxens species was recorded. For example *Lu. cavernicola*, *Ev. spelunca* and *Sc. sordellii* have a significant number of specimens captured, and more than 90% of the specimens of these species were registered in the cave. Thus, these species may be adapted to live in this environment, but they also depend, in part, on the energy source from the external environment.

*Evandromyia spelunca* is a recently described phlebotomine species of the *cortelezzii* complex described from this cave [12]. This species shows evidence of adaptation to the cave environment since it was captured in this study, in all months and was the second most abundant species in the cave.

A relevant data found in this study refers to the number of specimens collected in the first trap (Cave 01) inside the cave, and the trap of the cave entrance, representing 44% of the total sandflies collected in the study. These results corroborate those of Poulson & White [6] who describe that the twilight zone (cave area nearest the entrance) has the largest and most diverse fauna. In the case of sandflies, this higher density found near the cave entrance may be related to the increased supply of food (vertebrate hosts and vegetation) associated with the possibility of refuge in this environment.

The comparison of the ecological indexes demonstrates that the cave area and the surrounding area had a very similar profile (Table 3). The data of the ecological index in the environments studied demonstrate that each index should be analyzed according to the characteristics considered to generate them as different indexes cover different aspects of the species composition in an area. The Shannon’s index assigns greater value to rare species and Simpson’s index, little value. These characteristics of the indexes could explain the findings of our work. This data is corroborated by other researchers in ecology of insects that argue about the necessity of using different parameters/indexes for studying the structure of insect communities in different areas [43].

The results presented demonstrated a similar diversity and density of sandflies in the two environments. This data corroborate other studies which showed that the insect diversity and density of sandflies in caves can be equal to or greater than those found in the forest [21,22,41].

The human-induced environmental changes can affect the structure of insects communities, considering that cave areas and its surroundings require that two aspects are approached: entomological fauna and surveillance. Few studies have examined the ecological features of the sand fly populations in Brazil, especially studies that consider the ecological indexes and the faunistic composition [41,44,45] The ecological indexes analyzed with the faunal composition allow for more reliable analyses from different environments, since it considers both the present species and its frequency in the surveys as well as the abundance of specimens per species [45].

This point is exemplified in the present study, because, despite the similarities of the cave and surrounding areas considering the diversity, evenness and richness indexes, the analysis of the faunistic composition considering the abundance of specimens per species has demonstrated a statistically significant difference between these two areas (Figure 2). The result of this analysis could be explained by behavioral differences between the most abundant species of the two environments. The adaptation to the cave environment may be related to specializations developed by these insects, as it has been observed for other animals adapted to the cave environment.

Other recent published study describes the hourly activity of sandflies captured over 24-hours in the same cave [46]. With this data, it became clear that some species presented differences in the circadian cycle and, consequently, a different period of activity compared with sandflies trapped in the external environment. An example, *Lu. cavernicola*, demonstrated that, in fact, these adaptations may lead to different behavior and, consequently, to a faunal composition, distinct in the two environments, with some species more adapted to the aphotic zone.

In a similar study conducted in Serra da Bodoquena, in forest and caves areas, it was observed that the abundances of the species in the forest also differed from those found in the caves of the region [22].

Our data suggest that while the diversity of the two environments are similar, most species are classified as troglophiles and, possibly, use the cave during the lifecycle.

The study of seasonality of sandflies correlated with climatic variables (relative humidity and temperature) showed interesting results. When we analyzed the density of sandflies captured over 24 months in both environments, with the exception of the months of August and November, a larger number of sandflies was captured inside the cave. The correlation of climatic variables with sandflies densities in each month revealed that in these two months, the relative humidity reached peaks and the values recorded were similar to the cave humidity values. These peaks of humidity found in these two months in the surrounding area (outside cave) were correlated with the occurrence of rainfall days before of the catch. However, this was only an observation since it was not possible to collect precipitation data for subsequent inclusion in this study. As well, other data such as wind speed, which can influence the density, richness and even the successful in the collections.

This analysis suggests that the relative humidity may influence the density of captured sandflies in the surrounding environment studied. The largest number of collected sandflies within the cave can be due to the slight variation of climate variables in this environment. For example, the air humidity presents low amplitude and high percentage in all seasons of the year. This argument is reinforced when considering that the northern region of Minas Gerais has dry and warm climate most of the year. Deane and Deane [47] investigated seasonal influences on *Lu. longipalpis* and found that the increased humidity during the rainy season can directly promote the proliferation and survival of this sand fly species. Other studies, carried out in similar environments to our surroundings, demonstrated that sandflies are usually found in greater numbers during warm and humid months [31,34,48,49].

However, it is necessary to consider another fact when analyzing the relation between relative humidity and density of collected sandflies in both environments. High humidity was also recorded in September and, especially, in October. However it was not observed as well as in the months of August and November, the highest densities of sandflies in the external environment. This result can be explained because of the larger exposure to the weatherproof of traps in the surrounding environment, which makes the collection success it is not equal to the cave collection success on rainy days. As previously mentioned, the precipitation data were not collected, but in both months, September and October occurred rains in at least one of the days of exposure (observation data), which may explains these results.

Regarding these data on cave, it can be considered an auspicious environment to sandflies, for instance, for sandflies feeding, since the insects can find vertebrate species which serve as food source both at day time and the twilight. This environment can be sandflies shelter or refuge in view of the stability of climate variables.

The presence of *Leishmania* vector species, such as: *Lu. longipalpis*, *Ny. intermedia*, *Ny. neivai*, *Ny. whitmani*, *Mg. migonei*, deserves to be highlighted.

*Lutzomyia longipalpis*, the main vector of *Leishmania infantum* in America represented 2% of the total sandflies captured. This species was more frequent in the surrounding area. It was also registered in the cave environment accounting for 20% of *Lu. longipalpis* captured specimens. *Ny. intermedia* was the second more abundant species, considering the whole number of collected specimens and the more abundant species in the surrounding environment. *Ny. whitmani* was the second more abundant species in the surrounding. *Ny. intermedia* and *Ny. whitmani* were found in the cave, and are the main species involved in the transmission cycle of *Leishmania (Viannia) braziliensis* in Brazil [50]. *Ny. neivai* was captured in smaller numbers, but it is important to point out that this species is also part of the transmission of *Le. braziliensis* in several regions of Brazil [51].

*Mg. migonei* accounted for 70% of total sand fly specimens caught in the cave and for 30% in the surroundings. The role of this sand fly in the transmission of *Le. braziliensis* was initially associated with foci of the disease in the Southeastern Region of Brazil. Currently, the presence of this species as a vector seems to have extended to the Northeastern Region. However, *Mg. migonei* is considered a secondary vector [49]. Some studies have reported the natural infection of this species by *Leishmania* parasites [52-54].

Regarding to our data, it becomes extremely important to monitor cave areas due to its ecological significance, including karsts areas. Lassance is one of the six cities that comprise Serra do Cabral. Serra do Cabral is an isolated mountainous complex, in the center-north of the state of Minas Gerais. State Park of Serra do Cabral was recently created for Environmental Protection Area Serra do Cabral and with it, the environmental exploitation of the region has been significant.

Other studies, in several Brazilian karsts areas, that aim at better understanding the cave environment fauna are essential to prevent the biological losses and to monitor areas that may present risks to visitors. The studies of sandflies in this environment are of great importance in view of its role in the transmission cycle of leishmaniases and its constant presence in caves.
